# Comparing Patient and Clinician Perceptions of Health-Related Quality of Life in Urinary Tract Infections

**DOI:** 10.1001/jamanetworkopen.2026.18822

**Published:** 2026-07-31

**Authors:** Jessica Howard-Anderson, Mersedes Brown, Rachel Korn, Julie Miller, Katherine Sutton, Felicia Ruffin, Sherry Burton, Engels N. Obi, Nireesha Sidduri, Alexandre H. Watanabe, Emre Yucel, Hayden B. Bosworth, Helen W. Boucher, Deborah Collyar, Sarah B. Doernberg, Megan Oakes, Bryce B. Reeve, Vance G. Fowler, Heather A. King

**Affiliations:** 1 Division of Infectious Diseases, Department of Medicine, Emory University School of Medicine, Atlanta, Georgia; 2 Department of Population Health Sciences, Duke University School of Medicine, Durham, North Carolina; 3 Department of Medicine, Duke University School of Medicine, Durham, North Carolina; 4 Merck & Co, Inc, Rahway, New Jersey; 5 Center of Innovation to Accelerate Discovery and Practice Transformation, Durham Veterans Affairs Health Care System, Durham, North Carolina; 6 Duke Clinical Research Institute, Duke University School of Medicine, Durham, North Carolina; 7 Division of General Internal Medicine, Department of Medicine, Duke University School of Medicine, Durham, North Carolina; 8 Tufts University School of Medicine and Tufts Medicine, Boston, Massachusetts; 9 Patient Advocates in Research, Danville, California; 10 Division of Infectious Diseases, Department of Medicine, University of California School of Medicine, San Francisco

## Abstract

**Importance:**

There is limited research comparing patient- and clinician-reported health-related quality of life (HRQOL) in patients with infections.

**Objective:**

To compare what patients with complicated urinary tract infections (UTIs) describe about their HRQOL with the perceptions of their treating clinicians.

**Design, Setting, and Participants:**

This qualitative study used semistructured interviews of hospitalized patients with complicated UTI and clinicians who cared for them for at least 2 days at 2 academic hospitals in the southeastern US. The interviews were conducted between February 7 and May 26, 2023, with the analysis completed between May 1, 2023, and September 3, 2024.

**Main Outcomes and Measures:**

The main outcome was how clinician perceptions and patient experiences and descriptions aligned and diverged for different aspects of patient HRQOL. Directed content matrix and team-based qualitative techniques were used to facilitate analysis and presentation.

**Results:**

Interviews were conducted with 12 patients (median [IQR] age, 70 [60-78] years; 7 male [58.3%]) and 16 clinicians (median [IQR] age, 35 [31-43] years; 9 female [56.3%]). Four key concepts were identified: (1) the full range of impact of complicated UTI symptoms on patient functioning was not consistently recognized by clinicians, (2) clinicians’ perceptions of HRQOL were influenced by patients’ medical conditions and concerns about misattribution of symptoms to a UTI, (3) different terms and level of detail were used by patients and clinicians to describe HRQOL associated with complicated UTI, and (4) clinicians believe that HRQOL is important but approach their role in addressing HRQOL with varied experience and comfort.

**Conclusions and Relevance:**

This qualitative study of patients and their treating clinicians found important differences in how patients and clinicians perceive and describe the impact of complicated UTI on patient HRQOL. Understanding how patient and clinician perceptions of HRQOL diverge may help with the design of patient-centered outcomes for clinical trials and identify areas for enhanced patient-clinician communication.

## Key Points

**Question:**

How do patient-reported and clinician perceptions of health-related quality of life (HRQOL) compare in patients with complicated urinary tract infections (UTIs)?

**Findings:**

In this qualitative study of 12 hospitalized patients with complicated UTI and 16 of their treating clinicians, clinicians consistently did not recognize the full impact of complicated UTI symptoms on patient functioning and were more focused on medical factors and the expected trajectory of recovery.

**Meaning:**

These findings suggest the need to develop specific HRQOL tools that can be directly administered to patients in antibacterial trials to truly capture the patients’ experiences and better understand the effectiveness of new antibiotics.

## Teaser

This qualitative study compares perceptions between patients with complicated urinary tract infection and their treating clinicians about the patients’ health-related quality of life (HRQOL).

## Introduction

Complicated urinary tract infections (UTIs), including pyelonephritis and UTIs occurring in individuals with genitourinary structural or functional abnormalities, accounted for almost 2.5 million emergency department visits in the US from 2016 to 2018.^1,2,3^ Patients with complicated UTI frequently require hospital admission and are more likely to have antibiotic-resistant organisms than those with uncomplicated UTIs. Additionally, complicated UTI significantly contributes to the global health care burden.^2,4^

Complicated UTI is also one of the most common infectious disease syndromes studied in anti-infective registration trials. The US Food and Drug Administration (FDA) has advocated that anti-infective trials should strive to incorporate the patient voice and capture how patients feel and function to more comprehensively assess the efficacy of a new drug.^5^ To meet this goal, outcomes need to be developed that include assessment of patient health-related quality of life (HRQOL). However, the optimal method and tools for evaluating HRQOL in complicated UTI and other common infectious disease syndromes are unknown.^6,7,8,9^ Importantly, we need to understand whether clinician assessments of HRQOL could be used as a surrogate for patient self-report.

Research outside of infectious diseases has revealed a discordance between clinician and patient perceptions about the impact of medication adverse effects and physical symptoms on HRQOL.^10,11^ Experiences related to pain, fatigue, and the emotional impact of an illness are particularly difficult for clinicians to recognize and evaluate.^10,12,13^ In infectious diseases research, few studies have compared patient and clinician perceptions of HRQOL. A 1999 study in patients with HIV showed that clinicians were more likely to underreport symptoms and their severity compared with patients’ self-report.^14^ No published studies to date have compared patient reports and clinician perceptions of HRQOL for bacterial infections in general or complicated UTI in particular. In this study, we aimed to enhance the development and administration of HRQOL assessments in registrational complicated UTI trials by interviewing patients with complicated UTI and comparing their self-reported HRQOL experiences with the perceptions of their treating clinicians.

## Methods

### Study Design

In this qualitative descriptive study,^15,16^ we conducted semistructured, individual interviews (concept elicitation) with patients with complicated UTI and their treating clinicians from February 7, 2023, through May 1, 2023. The Duke University Health System Institutional Review Board approved this study. All participants provided written informed consent, which included permission to record and transcribe interviews and to report deidentified data; they were also compensated for their time. This study followed the Standards for Reporting Qualitative Research (SRQR) reporting guideline.^17^

The interview guide was developed for this study based on the Wilson and Cleary model of HRQOL. Interviews were conducted 10 to 20 days (patients) and 5 to 30 days (clinicians) after the qualifying urine culture collection. Interview time points were selected to align with the FDA guidance on developing novel anti-infectives for complicated UTI.^3^

### Participants and Setting

We prospectively enrolled patients from 2 hospitals in an academic health care system in the southeastern US. Patients were eligible for inclusion if they (1) had a quantitative urine culture yielding more than 100 000 colony-forming units/mL of bacteria; (2) met the recommended FDA inclusion criteria for complicated UTI (including ≥2 of the following signs or symptoms: chills, rigors, or fever [eg, oral temperature >38 °C]; flank or pelvic pain; nausea or vomiting; dysuria, urinary frequency, or urinary urgency; or costovertebral angle tenderness on physical examination)^3^; and (3) were aged 18 years or older, comfortable participating in an interview in English, and had no evidence of cognitive impairment via medical record review. Patients with conditions recommended for exclusion by FDA guidance (prostatitis, ileal loop or conduit, kidney transplant, recent trauma to urinary tract)^3^ or those lacking phone access were not enrolled. If potentially eligible participants appeared to be acutely cognitively impaired due to a medical condition, study staff would reassess their medical records throughout admission and approach them for possible study enrollment once their cognition had improved. Among eligible patients, we used purposeful sampling to capture a range of experiences, ensuring representation by sex, race, and age.

Eligible clinicians for the study included attending physicians, resident and fellow physicians, and advanced practice clinicians who cared for enrolled study patients for at least 2 calendar days. Clinicians could only be enrolled and interviewed for 1 study patient. Interested and willing clinicians were consented using an institutional review board–approved REDCap (Vanderbilt University) electronic consent, a secure, web-based application used to collect data,^18,19^ and were contacted via email to schedule an interview.

### Data Collection

Following best practices for qualitative research, we aimed to conduct interviews with approximately 12 to 15 patients and their treating clinicians, which was sufficient for saturation and/or information power.^20,21^ Patient and clinician interviews were conducted virtually by 2 authors (M.B. and J.M.), who are trained qualitative researchers with multiple years of experience. Interviews were audio recorded and transcribed.

#### Patient Data Collection

Patient interviews were designed to last approximately 60 minutes (patient interview guide provided in eAppendix 1 in Supplement 1). During the interview, research staff first administered a 6-item screener^22^ to identify participants with cognitive impairment. If an enrolled participant had 2 or more errors on the cognitive screener, they were no longer eligible to complete the interview. Sociodemographic information was collected as part of the study interview. Data on race were abstracted by members of the research team (R.K. and F.R.) from the electronic medical record, using the medical record classifications (American Indian or Alaska Native, Asian, Black or African American, White, multiracial, other, not recorded). We considered race (as well as other sociodemographic characteristics) to capture a range of experiences in patients with complicated UTIs. Data on patient comorbidities, including the Charlson Comorbidity Index,^23^ were abstracted from the electronic medical record by trained research staff (R.K. and F.R.) using a standardized data collection tool.

The interview guide was informed by the Wilson and Cleary model of HRQOL,^24^ adapted into patient-centered language, and developed to better understand patients’ complicated UTI symptoms and how these symptoms impact different domains of HRQOL (physical, functional, emotional, cognitive, and social). Another goal of the interview was to ascertain patient relationships and experiences with clinicians and their care team.

#### Clinician Data Collection

Clinician interviews were designed to last approximately 20 to 30 minutes. The clinician interview guide was designed to correspond to the patient guide but focused on clinician perceptions of HRQOL for their specific enrolled study patient and assessed broader, general perceptions of clinician-patient interactions (clinician interview guide provided in eAppendix 2 in Supplement 1). Before the interview, consented clinician participants were sent an electronic survey by the research team to collect sociodemographic information, including race and information about prior training and education.

### Data Analysis

The data analysis was performed between May 1, 2023, and September 30, 2024. We sought to understand how clinician perceptions and patient experiences and descriptions aligned and diverged for different aspects of patient HRQOL. Directed content analysis and summary, matrix, and team-based qualitative techniques were used to facilitate analysis and presentation.^25,26^ We organized the transcript data based on interview guide questions. To ensure consistency across transcripts, 3 study team members (M.B., J.M., and K.S.) analyzed the first transcript individually and then met as a team to reconcile discrepancies and achieve a consensus. Additionally, 2 of the 3 analysts conducted the interviews, which enhanced the rigor and credibility of the data analysis. After achieving a consensus, the 3 study team members proceeded to analyze the remaining transcripts independently and continued to meet as a team throughout the analysis process to resolve any discrepancies. The same 3 analysts then separately summarized within and across patient-clinician dyads (1 patient and 1 treating clinician) or triads (1 patient and 2 treating clinicians). The team maintained a record of analytic decisions using word processing and spreadsheet software.

## Results

We interviewed 12 patients (median [IQR] age, 70 [60-78] years; 5 female [41.7%] and 7 male [58.3%]; 6 of Black or African American [50.0%] and 6 of White [50.0%] race) (Table 1) and 16 of their treating clinicians (median [IQR] age, 35 [31-43] years; 9 female [56.3%] and 7 male [43.8%]; 3 of Asian [18.8%], 1 of Black or African American [6.3%], 9 of White [56.3%], and 2 of unknown [12.5%] race; 1 clinician was multiracial [6.3%]) (Table 2), comprising 8 patient-clinician dyads (1 patient and 1 treating clinician) and 4 triads (1 patient and 2 treating clinicians) (eFigure in Supplement 1). The mean length of patient interviews was 44 minutes (range, 20-74 minutes). Seven patients (58.3%) had urologic surgery, spinal cord injury, or urinary obstruction or instrumentation (including catheterization) in the past 30 days. *Escherichia coli* was the most common organism identified (10 patients [83.3%]), and all patients received intravenous antibiotics for at least a portion of their treatment (Table 1).

**Table 1.** Characteristics of Interviewed Participants Hospitalized With Complicated UTIs (N = 12)

Characteristic	Patients, No. (%)
Age, median (IQR) [range], y	70 (60-78) [43-80]
Sex	
Female	5 (41.7)
Male	7 (58.3)
Race	
Black or African American	6 (50.0)
White	6 (50.0)
Education level	
Some high school or high school graduate or GED	5 (41.7)
Some college or vocational school	3 (25.0)
College graduate or postgraduate education	4 (33.3)
Marital status	
Never married	4 (33.3)
Married	5 (41.7)
Widowed	3 (25.0)
Employment status	
Working full time	1 (8.3)
Retired	7 (58.3)
Unemployed or unable to work due to disability	4 (33.3)
Annual household income, $	
10 000-49 999	6 (50.0)
50 000-69 999	2 (16.7)
≥80 000	3 (25.0)
Declined to answer	1 (8.3)
Length of admission, median (IQR), d	9 (6-10)
Underlying comorbidity^a^	
Corticosteroid use^b^	1 (8.3)
Insulin-dependent diabetes	4 (33.3)
Solid organ or hematologic cancer	3 (25.0)
Charlson Comorbidity Index, median (IQR)^c^	9 (7-12)
Urologic history^a^	
Prior urologic surgery	4 (33.3)
Spinal cord injury	2 (16.7)
Urinary tract obstruction^b^	2 (16.7)
Urinary tract instrumentation, including catheterization^b^	1 (8.3)
None of the above	5 (41.7)
Organism in urine culture	
* Escherichia coli*	10 (83.3)
Other^d^	2 (16.7)
Pyelonephritis	1 (8.3)
Inpatient antibiotic therapy	
Oral and intravenous	11 (91.7)
Solely intravenous	1 (8.3)
Antibiotics prescribed at discharge for complicated UTI	
Oral	6 (50.0)
Intravenous	1 (8.3)

Abbreviations: GED, General Educational Development; UTI, urinary tract infection.

^a^ Not mutually exclusive.

^b^ Within the past 30 days.

^c^ Scale of 0 to 39, with higher scores indicating more comorbid conditions.

^d^ Included *Pseudomonas aeruginosa*, *Enterobacter cloacae* complex.

**Table 2.** Characteristics of Clinicians Caring for Patients With Complicated UTIs (N = 16)

Characteristic	Clinicians, No. (%)
Age, median (IQR) [range], y	35 (31-43) [26-56]
Sex	
Female	9 (56.3)
Male	7 (43.8)
Race	
Asian	3 (18.8)
Black or African American	1 (6.3)
White	9 (56.3)
Multiracial	1 (6.3)
Unknown	2 (12.5)
Level of practice	
Advanced practice clinician	1 (6.3)
Hospitalist	10 (62.5)
Trainee (resident or fellow)	5 (31.3)
Specialty	
Internal medicine	13 (81.3)
Internal medicine–pediatrics palliative	1 (6.3)
Oncology	1 (6.3)
Not reported	1 (6.3)
Clinical experience, median (IQR), y	6 (3-13)
Clinician who first diagnosed complicated UTI^a^	
Yes	9 (56.3)
No	7 (43.8)

Abbreviation: UTI, urinary tract infection.

^a^ This question was intended to determine whether the clinician cared for the patient when they were first diagnosed with the complicated UTI as opposed to caring for the patient after the complicated UTI had been diagnosed.

The mean length of the clinician interviews was 21 minutes (range, 11-31 minutes). Of the 16 clinicians interviewed, 10 (62.5%) were hospitalists, 5 (31.3%) were trainees (residents or fellows), and 1 (6.3%) was an advanced practice clinician. Overall, clinicians had a median (IQR) of 6 (3-13) years of experience (Table 2).

We identified 4 key concepts through our qualitative analysis: (1) The full range of impact of complicated UTI symptoms on patient functioning was not consistently recognized by clinicians, (2) clinicians’ perceptions of HRQOL were influenced by patients’ medical conditions and concerns about misattribution of symptoms to a UTI, (3) different terms and level of detail were used by patients and clinicians to describe HRQOL impact of the complicated UTI, and (4) clinicians believe that HRQOL is important but approach their role in addressing HRQOL with varied experience and comfort. The first 3 concepts highlight patient-clinician differences. The fourth concept focuses on how clinicians assess HRQOL. Table 3 describes each concept in detail and includes illustrative quotes.

**Table 3.** Key Concepts Comparing Patient and Clinician Perceptions of HRQOL

Key concept	Description	Illustrative quotes^a^
1. Full range of impact of complicated UTI symptoms on patient functioning was not consistently recognized by clinicians.	Clinicians did not always perceive or contextualize the extent to which complicated UTI symptoms impacted patients. When discussing the impact of HRQOL in patients with complicated UTI, clinicians tended to focus on impact during hospital admission and not how the experience impacted patients before or after admission.	Exemplar 1: patient and clinician Patient: “Yeah, it kind of just was worrying me about just the symptoms that I had because I know nobody in the family has ever had illness like that and it’s uncommon for a man. I thought it was just the women had stuff like that and deal with it.…And that’s it in a nutshell, basically. It drained me probably physically and mentally, basically, because of being that I had to deal with a situation that I’ve never been in.” Clinician: “I’m not sure, exactly. I don’t want to make a guess how this exactly impacted him. I think like any other illness, any other infection, any other hospitalization, it can really set you back and put you mentally in a tough place because it’s like putting your life on hold until you get better.” Exemplar 2: patient and clinician Patient: “Not exactly brain fog for me, just tired.…I’m slower to react to what people are saying...I understand what they’re saying to me, but it just takes me longer to process it.…It’s a little better now. It’s kind of strange, one day I’ll feel pretty good, and then the next day I may not feel so good. It’s not a linear getting better—a little bit today and a little bit better tomorrow. It seems to be up and down.” Clinician: “I might have missed that if that was there, but it is often that patients experience brain fog or delirium, but I can’t say, precisely, if that was the case in this patient.” Exemplar 3: patient and clinician Patient: “At times, it’s been tense with my wife.…We live by ourselves and when you bring a third party, it changes everything. My daughters. So, first we had daughter #2 the first week. Then daughter #1 the second week. And then daughter #3 wanted to come down, but we decided no, we needed a break.” Clinician: “So I think that he has a loving, large family with his wife and 3 daughters. He had a daughter fly in from New York to help take care of him for everyone. She mentioned that she was cleaning out the refrigerator and helping them take care of the house and getting things tidied up and fixed when he was in the hospital.…So, I, yeah it just—it seemed to be a big stressor for maybe even more so than [patient] would be a stressor for his wife.” Exemplar 4: patient and clinician Patient: “I really didn’t feel like going anywhere [while experiencing the complicated UTI].…I didn’t feel like going out and socializing….I just didn’t have the strength. I’d been hurting like I was. A lot of pain, you know.” Clinician: “Assuming it was a UTI…I don’t think it would impact her too much. Maybe some of the side effects from antibiotics…she has to go to the bathroom a ton. Maybe that means she won’t socialize as much at the nursing home because she’s gotta find out where the bathroom is.”
2. Clinicians’ perceptions of HRQOL were influenced by patients’ medical conditions and concerns about misattribution of symptoms to a UTI.	Clinicians tended to interpret the complicated UTI in the context of other potentially complex health situations and frequently saw the complicated UTI as a secondary concern. Clinicians expressed concern about the ambiguity of diagnosing a complicated UTI and wondered about the true attribution of patient symptoms.	Exemplar 1: patient and clinician Patient: “My stomach hurt real bad in a cramp. Painful.…I knew it was something funny because it kept burning. It never stopped burning.” Clinician: “This misattribution of her global weakness to what was likely either pyuria versus asymptomatic bacteriuria at the time, and then us missing, or not responding to quickly enough, a diagnosis of a primary respiratory issue. I think that’s probably what affected her the most. Though, I would also say that other things that can affect patients is when we diagnose them with urinary tract infection for non-GU symptoms; that perpetuates in their mind what a urinary tract infection is for them.…Folks feel any sort of vague weakness or bizarre nonlocalizing symptom and because we have this culture of treating these folks with urinary infections, then caregivers, family, and the patient themselves end up believing that some sort of cystitis or GU infection is causing these other symptoms.” Exemplar 2: patient and clinician Patient: “Well, I’ll tell you one way [the UTI] impacted me.…I’m tryin’ to avoid havin’ it. I wish I knew what was causin’ it. ‘Cause one thing: it’s embarrassing.…I don’t know why I keep getting infections, unless it’s coming from my bladder.…I wish I could find out where it comes from.” Clinician: “I think another UTI makes her picture a little bit more complicated and her overall physical kind of burden was more with the pneumonia and the UTI.…I do feel like with her cancer background, plus the pneumonia, and the UTI on top, it was just multiple hits that…affect her physical function to be safe and functional at home.” Exemplar 3: clinician only Clinician: “Yeah. So, I think it’s the million-dollar question. As a hospital, we have IDSA guidelines that are like if the patient’s asymptomatic, they don’t have a UTI. Even if they have bacteria growing in their blood, multidrug resistant, whatever. It’s not a UTI. And then, you have the patient in front of you, and that’s when it becomes much more difficult. And so, for this patient, I would say likely not a UTI for him. Very likely. I would put him less than a couple of percent change. But, in other cases, we have patients who come in who are altered, and they get fluids, and antibiotics, and all these other things, and they get better, but they never had symptoms. And, you’re like well, they got better with antibiotics, but was it the fluids that got them better? Was it being out of home? And, it’s always a difficult conversation and a difficult diagnostic dilemma where a lot of hospitals are dealing with it in such different ways.…So, I know we overdiagnose it. But, it’s really hard when there’s a patient in front of you. It’s like is this the overdiagnose or is this the right diagnosis?”
3. Different terms and level of detail were used by patients and clinicians to describe HRQOL impact of the complicated UTI.	Patients tended to be more detailed when describing their ideal HRQOL post discharge, whereas clinicians provided more general statements. Clinicians used terms, such as goals of care and return to baseline, which may not be familiar language for patients; this could represent an opportunity for enhanced patient-clinician communication.	Exemplar 1: patient and clinician Patient: “Getting back to bein’ normal. Gettin’ back to doin’ some of my activities I enjoy.…Get on the farm, go on with my life, and yeah, just looking across the fields looks good, but I like to get out in them too, just walkin’.” Clinician: “[Good HRQOL is] being catheter-free.…We treated his vasculitis, so he’s requiring less and less of these medications that can cause urine retention. So, I’m hoping that with this, he will not require catheterization down the road.” Exemplar 2: patient and clinician Patient: “I can enjoy myself better. I can move around. I can get to the point where I feel like being myself again. I’d like to get back to cooking.” Clinician: “I mean number one would be getting out of the hospital…that’s like the first step towards a more reasonable quality of life. I would assume for her…she would expect a resolution of dysuria prior to leaving the hospital. Or if the dysuria came back, then that would be a sign that the treatment had failed. I think this’s a common assumption that folks make despite that a lot of UTIs can recur if they are true UTIs.” Exemplar 3: patient and clinician Patient: “[G]etting back to livin’ my regular life…communicating with people,…that knew I was sick, family members because they said I sounded bad....They say I’m sounding a…bit better now....I’ve been…cautious about that, and…not trying to do as much till I feel like I’m back to my regular self again.” Clinician: “I would imagine the quality of life would be not having symptoms again. Not needing to come back to the hospital for anything.” Exemplar 4: clinician only Clinician: “Super important for any clinician to be aware of the patient’s goals. I think it’s often kind of disregarded or made secondary, tertiary, quaternary status. We have what’s seemingly more pressing medical goals to achieve, but for the patient, quality of life may be their most important goal, and in elderly, sick patients, it’s very important to discuss those goals upfront and have an idea of what the patient wants to achieve in terms of their goals of care while they’re inpatient.” Exemplar 5: clinician only Clinician: “I’m hopeful that he gets back to baseline and it really doesn’t overall impact him…but I mean, I have concerns because he’s gonna be very prone to them—to UTIs—and he’s got a lot of reasons for his.…And everything’s gonna be a complicated UTI based on both his anatomy and all the things we have sticking into him.”
4. Clinicians believe that HRQOL is important but approach their role in addressing HRQOL with varied experience and comfort.	There are a variety of ways in which physicians view HRQOL as part of their scope of practice. Clinicians with limited experience focused on ensuring that the infection was treated so that the patient could be discharged home. Clinicians with more experience tended to address follow-up plans, including providing education on prevention or referrals to improve activities of daily living.	Exemplar 1: clinician only Clinician: “I think as physicians, it’s our job to, of course, know how to manage and treat things that are physically affecting patients, but quality of life is still related to that. Because we treated the UTI, but she wasn’t up and doing things she used to do. Then that could lead to her having to come back or be in the hospital longer because she’s getting weaker and not healing or getting back to her baseline kind of functional status. So, I think it’s important for us to consider all those things. And then she could get depressed if she’s not back to her quality of life. So, I feel like it’s all related and something for us to be aware of as well. But kind of was a facilitator role to start these conversations and reach out to different people that would help us like physical therapy, occupational therapy, case management, etc.” Exemplar 2: clinician only Clinician: “I’ll be very honest. I don’t think I’ve thought about that enough. But I do think that is something that we could be a bit more cognizant of.…I do think addressing the impact on quality of life is an important thing.” Exemplar 3: clinician only Clinician: “I usually don’t address the quality of life. Because the expectation I think both on the patient’s part and my part would be that they would return to their previous quality of life. However, if I see risk factors, say a patient with chronic debility, malnutrition, dementia. Those risk factors for taking a step down and not getting back up to their quality of life, then I will have that conversation with the patient and the family that that’s an expectation that they may not get back to it. Maybe with lots of physical therapy and a lot of intensive medical care, but I always make the expectation that there could be a drop in quality of life after a major hospitalization, whether or not it’s UTI or not.”

Abbreviations: GU, genitourinary; HRQOL, health-related quality of life; IDSA, Infectious Diseases Society of America; UTI, urinary tract infection.

^a^ These selected quotes represent exemplars from 7 different patients and 11 clinicians (2 patients and 1 clinician are used in multiple concepts).

### Key Concept 1

For key concept 1—the full range of impact of complicated UTI symptoms on patient functioning was not consistently recognized by clinicians—patients discussed many domains of functioning that were affected by the complicated UTI and associated symptoms. Their answers included descriptions of complex emotions, including worry and uncertainty, and examples of how their illness added stress to their family or loved ones. In addition to the expected physical pain or discomfort associated with a complicated UTI, patients also expressed issues with fatigue, cognitive functioning, and a decreased desire to socialize. While clinicians acknowledged that the complicated UTI likely impacted HRQOL, they had difficulty describing and understanding the details of how patients were affected and the depth of the impact on HRQOL, particularly related to emotional and cognitive functioning. Commonly, clinician answers were phrased as educated guesses or assumptions about what the patient might be experiencing based on medical knowledge or other objective details about the case.

### Key Concept 2

For key concept 2—clinicians’ perceptions of HRQOL were influenced by patients’ medical conditions and concerns about misattribution of symptoms to a UTI—clinicians tended to focus on how the complicated UTI was related to the patient’s acute medical conditions or other comorbidities, often minimizing or questioning the impact of the complicated UTI. Clinicians expressed diagnostic uncertainty and wondered whether the patients’ symptoms should all be ascribed to the complicated UTI. They discussed that patients could have bacteria present in a urine culture (ie, bacteriuria) but not necessarily have a complicated UTI, which makes diagnosing complicated UTI challenging and even harder to explain to patients and their caregivers.

In contrast to clinicians, patients did not openly express ambiguity or concern about the correct diagnosis. They viewed the complicated UTI as a distinct medical problem that caused them burdensome, impactful symptoms that should be treated. Patients expressed a desire to understand how to prevent a complicated UTI from happening again.

### Key Concept 3

Similar to key concept 1, for key concept 3—different terms and level of detail used by patients and clinicians to describe HRQOL impact of the complicated UTI—patients tended to elaborate and provide more detail on issues related to HRQOL. They described activities that they hoped to be able to do when they returned home or recovered from their illness. Clinicians focused on medical terms and key treatment objectives, including symptom improvement, removal of the urinary catheter (if applicable), and hospital discharge. Clinicians used medical jargon, such as return to baseline and goals of care, which may not be understood by patients and may limit the discussion of patients’ expectations and desires during their recovery period.

### Key Concept 4

For key concept 4—clinicians believe that HRQOL is important but approach their role in addressing HRQOL with varied experience and comfort—while clinicians acknowledged that HRQOL is an important aspect of patient care, the degree to which they felt ownership of this or saw it as their responsibility varied by clinician. Several clinicians focused on symptom resolution, prioritizing patients feeling well enough to go home and recover; others mentioned having a more active role in facilitating resources after discharge or providing expectations of how HRQOL may be impaired after an illness. Clinicians with more experience or comfort with addressing HRQOL mentioned addressing patients’ personal goals and taking additional steps to improve patients’ functioning after discharge.

## Discussion

This qualitative study identified and described key concepts comparing patient and clinician perceptions of HRQOL in patients with complicated UTI. Clinicians often did not describe or identify the entire spectrum of impact of the complicated UTI and associated symptoms on patients’ functioning, particularly the emotional and cognitive aspects. Clinicians, perhaps unsurprisingly, tended to focus on resolution of classic complicated UTI symptoms (eg, dysuria, flank pain) and prioritized determining whether the patient’s diagnosis was accurate. Patients discussed their desire to feel better and prevent symptom recurrence. Additionally, the language used by clinicians to describe HRQOL was different from the language used by patients. Interviews with clinicians revealed that they had varied levels of comfort and experience addressing HRQOL and differing beliefs regarding their role in improving patients’ HRQOL.

We are not aware of any other studies comparing clinician and patient perceptions of HRQOL in complicated UTI; however, our findings are similar to prior urologic studies showing that physicians significantly underestimate the burden or impact of lower urinary tract symptoms. This underestimation was most often pronounced for the social and emotional domains.^11,13^ While our analysis identified differences between patients’ perceptions and those of their treating clinicians, it is also important to acknowledge the similarities between the patients’ and clinicians’ viewpoints. Clinicians consistently mentioned that HRQOL was an important component of their patients’ health and acknowledged that social limitations could occur based on the patients’ complicated UTI.

This study supports the need for an HRQOL assessment completed independently by patients to fully capture the impact of HRQOL and to monitor changes. While previous work has shown that patients with uncomplicated UTIs have poor HRQOL, this has been less commonly studied in patients with complicated UTI.^6,27,28^ A physician assessment may be able to focus on objective complicated UTI symptoms and discharge planning, but without patient self-report, the true patient voice and full experience related to the complicated UTI could be lost. Additionally, data from the cardiology literature have shown that patient-reported HRQOL may be a better predictor of clinical outcomes than a clinician-assessed functional score.^29^

Our analysis also revealed differences in the terms and vocabulary used by patients and clinicians to describe HRQOL and treatment plans. Future work should seek to better understand these differences, including among different clinicians treating the same patient, to help enhance patient-clinician communication. Additionally, while not addressed in this study, many patients with complicated UTI are older adults and have cognitive impairment, which may impact communication with clinicians about their HRQOL. If clinicians and patients (or patients’ caregivers) are better able to express their concerns and expectations for recovery with a shared understanding, this may bridge gaps in care and lead to additional improvements in HRQOL.

### Limitations

Our study had several limitations. We only included patients from a single academic health care system and 2 racial groups, as well as interviewed patients in English. Thus, our work may not represent the experiences of other patients from different care settings, geographic areas, races, or cultural groups. Additionally, the definition of complicated UTI is not standardized and may differ from patient enrollment in clinical trials.^30,31^ Since we focused on outcome development for novel anti-infective agents, we used the recommended inclusion and exclusion criteria described in current FDA guidance for complicated UTI trials.^3^ The FDA inclusion criteria require systemic symptoms but, notably, do not include a requirement for abnormal genitourinary anatomy. In our analysis, 5 patients did not have a documented abnormality of the urinary tract or recent instrumentation; therefore, in other studies, it is possible that such patients may be classified as having uncomplicated UTI. However, all study patients were hospitalized with a high proportion of comorbid conditions, meaning that most may have been treated as having a complicated UTI. Our interview time points also were chosen to align with FDA trial guidance; however, using this relatively short follow-up period may mean that we did not capture long-term HRQOL in patients with complicated UTI. Finally, we acknowledge that because we determined patient eligibility in part via medical record review, this does not completely mimic clinical trial procedures.

## Conclusions

This qualitative study of patients and their treating clinicians identified critical differences in how patients and their treating clinicians describe and perceive HRQOL. Clinicians did not consistently recognize the full impact a complicated UTI could have on patient functioning and were more focused on medical factors and the expected trajectory of recovery. Future work should develop and use specific HRQOL assessments that could be administered to patients in clinical trials to truly capture the patients’ experience and better understand the utility and effectiveness of new antibiotics.

## Notes

Supplement 1. **eAppendix 1.** Patient Concept Elicitation: Semistructured Qualitative Interview Guide **eAppendix 2.** Clinician Concept Elicitation: Semistructured Qualitative Interview Guide **eFigure.** Flowchart of Patient and Clinician Enrollment

Supplement 2.
Nonauthor Collaborators

Supplement 3.
Data Sharing Statement
