# Supplementary material for: Comparing Patient and Clinician Perceptions of Health-Related Quality of Life in Urinary Tract Infections
Source: JAMA Netw Open. 2026 Jul 31;9(7):e2618822. doi: 10.1001/jamanetworkopen.2026.18822 (PMC13428275; doi:10.1001/jamanetworkopen.2026.18822)
Supplement: Supplement 1. — eAppendix 1. Patient Concept Elicitation: Semistructured Qualitative Interview Guide eAppendix 2. Clinician Concept Elicitation: Semistructured Qualitative Interview Guide eFigure. Flowchart of Patient and Clinician Enrollment [file jamanetwopen-e2618822-s001.pdf]

Supplemental Online Content

Howard-Anderson J, Brown M, Korn R, et al; Antibacterial Resistance Leadership Group. Patient and clinician perceptions of health-related quality of life in urinary tract infections. *JAMA Netw Open*. 2026;9(6):e2618822. doi:10.1001/jamanetworkopen.2026.18822

eAppendix 1. Patient Concept Elicitation: Semistructured Qualitative Interview Guide

eAppendix 2. Clinician Concept Elicitation: Semistructured Qualitative Interview Guide

eFigure. Flowchart of Patient and Clinician Enrollment

This supplemental material has been provided by the authors to give readers additional information about their work.

© 2026 Howard-Anderson J et al. *JAMA Network Open*.

eAppendix 1. Patient Concept Elicitation: Semistructured Qualitative Interview Guide

Interview Questions

1. **What caused you to seek medical help/attention?**
    a. Probe: What symptoms, or feelings, did you have that made you seek medical help? (i.e. fever, chills, frequent urination/urination urgency, pain or burning with urination, lower abdominal pain, flank/back pain, nausea/vomiting, confusion, etc.)
    b. Let's think about each of the symptoms you just mentioned. How severe did you think (fill in with symptom) was/were at that time? [Ask about each symptom mentioned]
    c. How long did you have these symptoms before you decided to get medical attention? [Probe: How long did each symptom last?]
    d. Did these symptoms start fairly quickly or come on slowly?
    e. At which point, if any, were you made aware that you have a UTI?

2. **Please describe the impact that your UTI symptoms and care have had on your body (or physically).**
    a. Which symptoms were the most bothersome? (i.e. frequent urination, burning sensations, fever etc)
    b. How long did these symptoms impact your body? Are these symptoms still happening?
    c. Did you experience any pain as a result of your UTI? How would you describe the pain you experienced as a result of your UTI, if any? (i.e., does it come-and-go or is it constant? sharp, dull, lingering, location of pain [pelvic, upper/lower back pain], etc.)?
        Probe: How has your pain related to your UTI changed over time?
        Probe: Are you still experiencing pain related to your UTI? If so, can you describe that pain you are feeling now?

3. **Please describe how your UTI symptoms and care have impacted your ability to do normal day-to-day activities (i.e., functionally).**
    a. What activities have been most impacted by your UTI symptoms?
    b. How, if at all, did you change your normal daily activities as a result of your UTI and care(i.e., dressing, recreational activities (exercise), work, etc.)? Please describe specific activities that had to be changed.
    c. If working, did you take time off? Have you returned to work yet?

4. **Please describe how your UTI symptoms and care have impacted you emotionally, or how you feel.**
    a. What words come to mind that describe your feelings about your experience with UTI? (examples: surprise, anxiety, anger, joy, fear, sadness, calm, loneliness, etc.)
    b. Were there certain symptoms or experiences that caused a change in your feelings? Please describe them, and if or how this changed over time.
    c. How have you been coping with these feelings?

5. **Please describe the ways in which your UTI symptoms and care have impacted your ability to think clearly.**
    a. Probe: For example, has your memory, ability to learn, concentrate, communicate, and/or make decisions been impacted by your symptoms?
    b. When did you first notice these changes to your ability to think clearly?
    c. Has your ability to think clearly changed over time?
    d. Are you still experiencing these symptoms?

© 2026 Howard-Anderson J et al. *JAMA Network Open*.

e. Specifically, did any change in your ability to think affect your feelings?
f. How did your ability to think impact your normal daily activities?
g. Did someone else who was with you have to inform you that you had difficulty thinking clearly or were out of it?

6. **As a result of this experience including all aspects of care, medical intervention, social support, have you noticed any changes in your relationships or social interactions?**
    a. Before your UTI symptoms began, how were your social relationships?
    b. Have you noticed a change in your social relationships as a result of your UTI symptoms? If so, what are the changes you notice?
        Probe: Are you more withdrawn in situations than in the past? Are you more reliant on others now?
        Probe: Conversely, do you feel that this experience has resulted in feeling more supported or having more contact with friends/family? Please explain.
    c. Probe only if hospitalized/in rehab, etc. - How do you think your time with friends and family would be different if you were back to your prior living situation?

7. **What symptoms are you (or were you) most interested in addressing with your care?**
    a. How do you feel about the quality of your care for your UTI?
    b. What have you liked best about your care? What would you like to change about your care or recommend for others?
    c. How manageable do you find your care to be for your lifestyle?
    d. What impact has your care had on your symptoms?
    e. What symptoms has your care most impacted?

8. **Thinking ahead, can you describe what having a good quality of life would look like to you after your UTI?**
    a. What changes would have to happen for you to feel like you have a good quality of life?

9. **How do you think your experience with this UTI will impact you overall?**
    a. How do you think this will impact your health in general in the future?
    b. Has this experience changed how would you seek help for new medical conditions? If so, please describe.

10. **Please describe your experiences and interactions with the medical team.**
    a. What things did you like about your experience with medical staff?
    b. What things would you change about your experience with medical staff?
    c. How well did your medical staff understand your experience?
    d. Has this experience changed your perception of healthcare with your provider?
    e. Please describe any specific differences between your UTI and any other conditions that you had treated?

11. **How comfortable did you feel asking your provider/NP/PA/medical professional questions about your UTI (diagnosis, care, etc.)?**
    a. How well did you understand the information given to you?
    b. Probe: If participant says they felt uncomfortable asking questions: Can you tell me more about why you felt uncomfortable asking questions?
    c. Do you feel your care was personalized to you?

© 2026 Howard-Anderson J et al. *JAMA Network Open*.

eAppendix 2. Clinician Concept Elicitation: Semistructured Qualitative Interview Guide

Interview Questions

1. Please describe how you became involved in the care of [Patient's Name] and what you know about their hospital admission.

2. What UTI symptoms did the patient describe/self-report? (e.g. at time of diagnosis. Or have clinician specify the time point they cared for them and ask about symptoms reported at that time point)

3. How do you think the UTI and subsequent treatment physically impact [Patient's Name] ability to do normal "day to day" activities?

4. How do you think this experience, including the UTI diagnosis and treatment, has impacted [Patient's Name] emotionally?

5. How do you think the UTI and treatment impact [Patient's Name] cognitive abilities?

6. How do you think the UTI and treatment impacted (or will impact) [Patient's Name] social relationships and experiences?

7. Can you describe what you think the patient would consider as being a good quality of life after this UTI?

8. What do you feel your role is as a clinician in terms of addressing health related quality of life concerns for patients?

9. Overall, how do you think your interaction was with [Patient Name]? To your knowledge did this differ with the rest of the medical team?

10. And finally, how do you think [Patient's Name] experience with this UTI will impact them overall? Is there any impact you think is important to mention that we did not previously discuss?

© 2026 Howard-Anderson J et al. *JAMA Network Open*.

eFigure. Flowchart of Patient and Clinician Enrollment

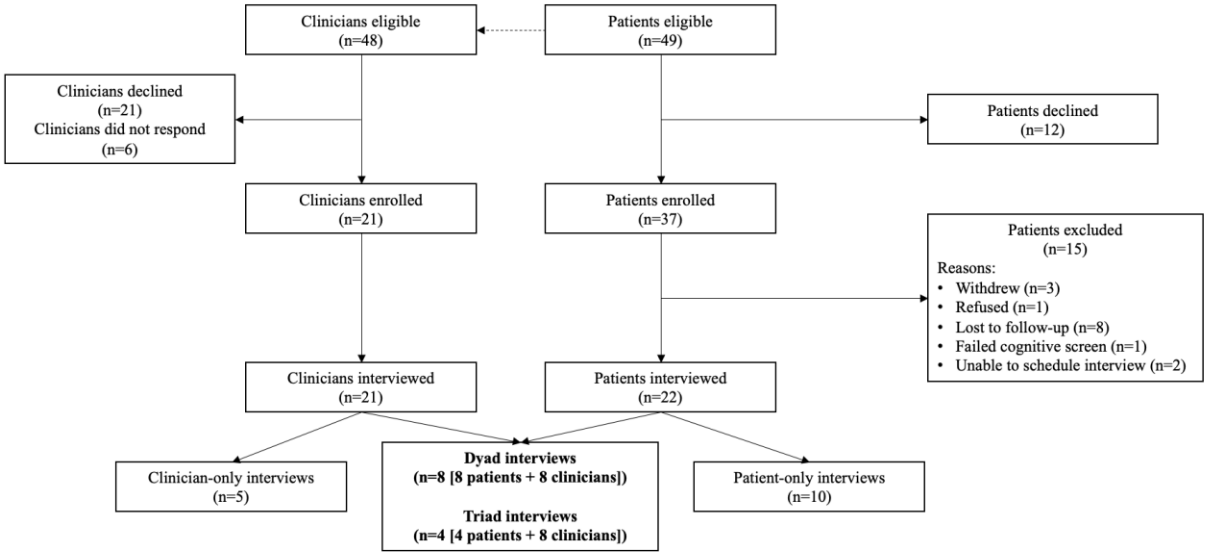

For this manuscript, we only analyzed and presented the results of the dyad and triad interviews (12 patients and 16 clinicians, in bold) because we were most interested in how clinician perceptions and patient experience/descriptions aligned and diverged for different aspects of patient health-related quality of life (HRQoL).

© 2026 Howard-Anderson J et al. *JAMA Network Open*.
