# Supplementary material for: Comparing Patient and Clinician Perceptions of Health-Related Quality of Life in Urinary Tract Infections
Source: JAMA Netw Open. 2026 Jul 31;9(7):e2618822. doi: 10.1001/jamanetworkopen.2026.18822 (PMC13428275; doi:10.1001/jamanetworkopen.2026.18822)
Supplement: Supplement 2. — Nonauthor Collaborators [file jamanetwopen-e2618822-s002.pdf]

Supplemental Online Content: Nonauthor Collaborators
*First name, last name, and suffix (if applicable) are required and will appear in PubMed.

*Group Name(s): Antibacterial Resistance Leadership Group							
***First Name and Middle Initial(s)**	***Last Name**	***Suffix (eg, Jr, III)**	Academic Degrees	Institution	Location (city, state/province, country)	Role or Contribution, eg, chair, principal investigator	Group (if more than 1 Group listed in the byline) and/or Subgroup (eg, Steering Committee)
Henry F.	Chambers						
Scott	Evans						
Vance	Fowler	Jr.					
Toshimitsu	Hamasaki						
Robin	Patel						
Heather	Cross						
Anthony	Harris						
Melinda	Pettigrew						
David	van Duin						
Helen	Boucher						
Clayton	Huntley						
Erica	Raterman						
Tamika	Samuel						
Kyung	Moon						
Kim	Hanson						
Yohei	Doi						
Thomas	Holland						
Tom	Lodise						
Ritu	Banerjee						
Sara	Cosgrove						
David	Paterson						
Ebbing	Lautenbach						
Maureen	Mehigan						
Sarah	Doernberg						
Sam	Perdue						
