# Supplementary material for: Comparing Patient and Clinician Perceptions of Health-Related Quality of Life in Urinary Tract Infections
Source: JAMA Netw Open. 2026 Jul 31;9(7):e2618822. doi: 10.1001/jamanetworkopen.2026.18822 (PMC13428275; doi:10.1001/jamanetworkopen.2026.18822)
Supplement: Supplement 3. — Data Sharing Statement [file jamanetwopen-e2618822-s003.pdf]

Data Sharing Statement

Data
Data available: Yes
Data types: Other (please specify)
Additional Information: Upon request, the authors will share more detailed information regarding the qualitative data and analysis, including the record of analytic decisions and more detailed analytic documentation.
How to access data: heather.king@duke.edu
When available: With publication

Supporting Documents
Document types: Other (please specify)
Additional Information: Upon request, the authors will share more detailed information regarding the qualitative data and analysis, including the record of analytic decisions and more detailed analytic documentation.
How to access documents: heather.king@duke.edu
When available: With publication

Additional Information
Who can access the data: Anyone requesting the data
Types of analyses: For any purpose
Mechanisms of data availability: After approval
